# VEGF-A-related genetic variants protect against Alzheimer’s disease

**DOI:** 10.18632/aging.203984

**Published:** 2022-03-28

**Authors:** Alexandros M. Petrelis, Maria G. Stathopoulou, Maria Kafyra, Helena Murray, Christine Masson, John Lamont, Peter Fitzgerald, George Dedoussis, Frances T. Yen, Sophie Visvikis-Siest

**Affiliations:** 1 IGE-PCV, Université de Lorraine, Nancy 54000, France; 2 Inserm, C3M, Team Control of Gene Expression (10), Université Cote d’Azur, Nice, France; 3 Department of Nutrition and Dietetics, School of Health Science and Education, Harokopio University, Athens 17671, Greece; 4 Randox Laboratories Limited, Crumlin, County Antrim BT29 4QY, United Kingdom; 5 Qualivie, UR AFPA laboratory, University of Lorraine, Vandoeuvre-les-Nancy, Lorraine, France

**Keywords:** Apolipoprotein E, LSR, VEGF-A, epistasis, Alzheimer’s disease, elastic net, machine learning, prediction

## Abstract

The Apolipoprotein E (*APOE*) genotype has been shown to be the strongest genetic risk factor for Alzheimer’s disease (AD). Moreover, both the lipolysis-stimulated lipoprotein receptor (LSR) and the vascular endothelial growth factor A (VEGF-A) are involved in the development of AD. The aim of the study was to develop a prediction model for AD including single nucleotide polymorphisms (SNP) of *APOE*, *LSR* and VEGF-A-related variants.

The population consisted of 323 individuals (143 AD cases and 180 controls). Genotyping was performed for: the *APOE* common polymorphism (rs429358 and rs7412), two *LSR* variants (rs34259399 and rs916147) and 10 VEGF-A-related SNPs (rs6921438, rs7043199, rs6993770, rs2375981, rs34528081, rs4782371, rs2639990, rs10761741, rs114694170, rs1740073), previously identified as genetic determinants of VEGF-A levels in GWAS studies. The prediction model included direct and epistatic interaction effects, age and sex and was developed using the elastic net machine learning methodology.

An optimal model including the direct effect of the *APOE e4* allele, age and eight epistatic interactions between *APOE* and *LSR*, *APOE* and VEGF-A-related variants was developed with an accuracy of 72%. Two epistatic interactions (rs7043199*rs6993770 and rs2375981*rs34528081) were the strongest protective factors against AD together with the absence of *ε4 APOE* allele. Based on pathway analysis, the involved variants and related genes are implicated in neurological diseases.

In conclusion, this study demonstrated links between *APOE*, *LSR* and VEGF-A-related variants and the development of AD and proposed a model of nine genetic variants which appears to strongly influence the risk for AD.

## INTRODUCTION

Alzheimer’s disease (AD) is a chronic, neurodegenerative disorder and the most common cause of dementia, characterised by mental and functional impairment [1]. It is associated with accumulation of neuronal amyloid plaques and early lesions primarily in hippocampus. It is estimated that AD prevalence doubles every 5 years in individuals over the age of 65. The World Health Organization has stated that AD constitutes a growing universal public health issue with enormous consequences on both individuals and communities [2].

AD severely affects the life of the patient, causing dependency, disability and subsequent fatality [2, 3]. There are two basic types of AD: a) familial or early onset AD which is responsible for more than 5% of the disease incidence and; b) sporadic or late onset AD that accounts for 79% of the disease burden. Late onset AD is highly heritable and it is etiologically heterogeneous originating from a mixture of multiple genetic and environmental risk factors.

Some of the genes that have been associated with the risk of sporadic AD include the *ABCA7*, *APOE*, *BIN1*, *CD2AP*, *CD33*, *CLU*, *CR1*, *EPHA1*, *MS4A4A/MS4A4E/MS4A6E*, *PICALM*, and *SORL1* genes [4]. More than 40 genes/loci have been associated with the risk of AD through the last 10 years, based on genome-wide association studies (GWAS) [5].

The most important genetic factor for AD is the Apolipoprotein E gene (*APOE*). The *APOE* gene codes for a 35 kDa glycoprotein, the apolipoprotein E (ApoE), which is strongly expressed in the brain [6]. There are three most common allelic variants in the *APOE* gene that alter the protein sequence leading to the formation of three different *APOE* isoforms: *APOE2* (cys112, cys158), *APOE3* (cys112, arg158), and *APOE4* (arg112, arg158) [6, 7] arising from 3 alleles, respectively, *ε2*, *ε3* and *ε4*. These alleles are associated with different ApoE roles [7]. The *ε4* allele is the strongest risk factor for late-onset AD [8, 9], due to its association with increased amyloid deposition and is a known risk factor for cardiovascular disease (CVD) [10]. Individuals with one *ε4* allele have a 2 to 3-fold elevated risk of developing AD, while those with two *ε4* alleles have about 12-fold increased risk compared to individuals who do not have the *ε4* allele. On the other hand, the *ε2* allele of the *APOE* gene appears to display a protective role, as it is associated with reduced risk for AD [7], but remains a risk factor for Type III hyperlipidemia [11]. Thus, this common polymorphism is an excellent candidate to study in the development of genetic risk prediction models for AD.

The human *APOE* gene is situated on the long arm of chromosome 19q13.1, an AD-associated zone as reported by GWAS [5]. The lipolysis-stimulated lipoprotein receptor (*LSR*) gene is also located in the same region and encodes the lipolysis-stimulated lipoprotein receptor (LSR) which recognizes ApoE as ligand [12]. As an ApoE receptor, LSR is involved in the process of managing and maintaining lipid balance in the peripheral and central nervous system [13, 14] Recently, our team identified significant epistatic interactions between two *LSR* gene single nucleotide polymorphisms (SNPs) and *APOE* in AD patients [12], namely the rs34259399 and the rs916147 SNPs. The former is located on exon 6 and the latter is located in a splicing junction between intron 5 and exon 6. Both these SNPs have been studied by our group and preliminary results indicate a functionality in terms of gene expression modification, while the rs916147 was also associated with lipids in a population of obese individuals (Yen et al., unpublished results).

The vascular endothelial growth factor A (VEGF-A) is also considered as a risk factor for chronic diseases, including AD. The VEGF family plays important roles in angiogenic regulation, neurogenesis and neuronal survival [15]. Although an inverse relationship has also been demonstrated [16], decreased levels of VEGF-A in serum and cerebrospinal fluid have been linked with increased risk for AD and cognitive impairment [17, 18].

Our group has focused on the study of VEGF-A for many years and has been involved in two GWAS by which ten genetic variants have been identified explaining more than 50% of the individual variability of VEGF-A levels [19, 20]. This exceptionally high percentage of variability explained by these variants makes them optimal target SNPs to be used in the candidate genes association studies as determinants of VEGF-A levels. In several previous studies we have demonstrated associations of these polymorphisms with intermediate phenotypes of CVD and other chronic diseases, such as autoimmune thyroid disease and depression [21–25], where VEGF-A is involved in several of their pathophysiology pathways. We have initiated and we are coordinating the Vascular Endothelial growth factor European Genomic Federation Consortium - VEGF Consortium (Sophie Visvikis-Siest coordinator, http://www.vegfconsortium.org) for the study of VEGF-A in chronic diseases and personalized medicine [26].

The precise detection of individuals at high risk for AD is very critical for early diagnosis and appropriate management enabling closer monitoring, enhanced care, as well as closer supervision of targeted risk factors-based interventions [27]. Even though late-onset AD is known to be a multifactorial disease with a strong genetic component, the use of common genetic variations identified in GWAS in disease prediction modelling has been of limited value so far, given that such polymorphisms explain a small relative risk and proportion of the underlying genetic contribution. Thus, it has been proposed that the predictive ability of models would be improved with the inclusion of true functional variants, the incorporation of epistatic effects and the combination with nongenetic biomarkers [28].

In the present study, a machine-learning predictive model for AD risk was developed, using a case-control population of late-onset AD patients and combining novel variants on candidate molecules for AD that have not been assessed before (VEGF-A related variants), as well as their epistatic interactions with known functional polymorphisms (*APOE* and *LSR*).

## RESULTS

The characteristics of the final population used in the analysis are presented in Table 1. The mean age of participants was 74.59 and 69.94 years old in patients and non-patients, respectively. Men constituted 37.36% of the control population, whereas they contributed to almost half in the cases group (i.e., 47.58%).

**Table 1:** Populations’ characteristics.

	**Age**		**Sex (male)**	
	**Mean**	**SD**	***n***	**%**
Controls (*n* = 182)	74.59	8.39	68	37.36
Patients (*n* = 145)	69.94	8.66	69	47.58

Abbreviation: SD: standard deviation.

The SNPs included in the analysis, their minor allele frequencies and their annotation on the genome are presented in Table 2. Since the rs114694170 SNP did not abide by the Hardy-Weinberg equilibrium law, it was removed from the analysis.

**Table 2:** Characteristics of the genotyped polymorphisms.

**Variants**	**Genes**	**All**		**Controls**		**Patients**	
		**MAF**	**HW *p*-value**	**MAF**	**HW *p*-value**	**MAF**	**HW *p*-value**
rs10761741	*JMJD1C*	0.45	0.77	0.47	0.77	0.44	0.77
rs6921438	*LOC100132354 and C6orf223*	0.42	0.43	0.41	0.43	0.42	0.43
rs7043199	*VLDLR-AS1*	0.24	0.90	0.26	0.90	0.23	0.90
rs6993770	*ZFPM2*	0.33	0.71	0.33	0.71	0.34	0.71
rs114694170	*MEF2C*	0.45	**0.00**	0.47	**0.00**	0.44	**0.00**
rs1740073	*POLR1C*	0.38	0.33	0.38	0.33	0.38	0.33
rs2375981	*KCNV2 and VLDLR*	0.47	0.26	0.44	0.26	0.49	0.26
rs34528081	*VEGF-A*	0.39	0.93	0.41	0.93	0.36	0.93
rs916147	*LSR*	0.37	0.29	0.38	0.29	0.36	0.29
rs34259399	*LSR*	0.14	0.76	0.12	0.76	0.15	0.76
rs4782371	*ZFPM1*	0.30	0.96	0.34	0.96	0.26	0.96
rs2639990	*ZADH2*	0.09	0.94	0.12	0.94	0.07	0.94
rs429358	*APOE*	0.22	0.77	0.22	0.77	0.22	0.77
rs7412	*APOE*	0.06	0.43	0.06	0.43	0.06	0.43

Abbreviations: MAF: Minor allele frequency; HW: Hardy-Weinberg.

**Figure 1:**
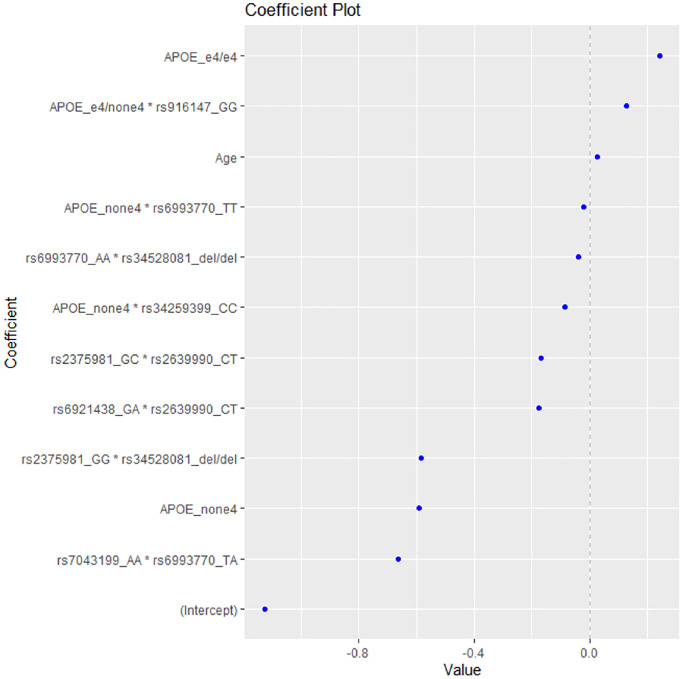
The coefficient plot of the EN model with the highest accuracy.

After applying the EN method to the data, the model with the highest accuracy included the variables presented in Figure 1. As expected, the presence of 2 *ε4* alleles (homozygotes *e4*/*e4*) was the first variable to be linked with increased risk for AD, while the absence of *ε4* allele was shown to be associated with decreased risk.

The epistatic interaction of *ε4* allele heterozygotes with the rs916147 variant of *LSR* gene was also associated with higher risk for AD, followed by age as the third risk factor for AD (Table 3).

**Table 3:** Coefficients of the prediction model.

		**Coefficient**
Risk factor variants	*APOE*_*ε4*/*ε4*	0.24
	*APOE*_*ε4*/non ε4*rs916147	0.13
	Age	0.03
Protective factor variants	rs7043199*rs6993770	−0.66
	*APOE*_non *ε4*	−0.59
	rs2375981*rs34528081	−0.58
	rs6921438*rs2639990	−0.18
	rs2375981*rs2639990	−0.17
	*APOE*_non ε4*rs34259399	−0.09
	rs6993770*rs34528081	−0.04
	*APOE*_non ε4*rs6993770	−0.02

The model included five epistatic interactions between VEGF-A-related variants, one APOE*LSR (rs34259399) interaction and one APOE*VEGF-A-related polymorphism interaction (for the rs6993770 SNP), associated with decreased risk for AD (Table 3). The strongest predictive factor of the model appears to be the interaction rs7043199*rs6993770, which decreases the risk of AD, thus playing a protective role, followed by the absence of the *e4 APOE* allele and the interaction rs2375981*rs34528081.

The accuracy of the model is 72% with a confidence interval CI = (0.6, 0.8) for 95%. The above accuracy has been calculated under a model with *P*-value: 0.009. The area under the curve (AUC) of the model is 81%, which indicates the detection of true positives (true cases predicted as cases) versus false positives (true controls predictive as cases).

Based on the annotation of the identified polymorphisms of the prediction model, we identified the following list of genes involved in AD risk: *VLDLR-AS1, APOE, KCNV2, ZADH2, C6orf223, LSR, ZFPM2,* and *VEGF-A*. After uploading the list to the IPA tool, the top five diseases found to be associated with the aforementioned were CVD, ophthalmic diseases, organismal injury and abnormalities, neurological diseases, and cancer.

**Figure 2:**
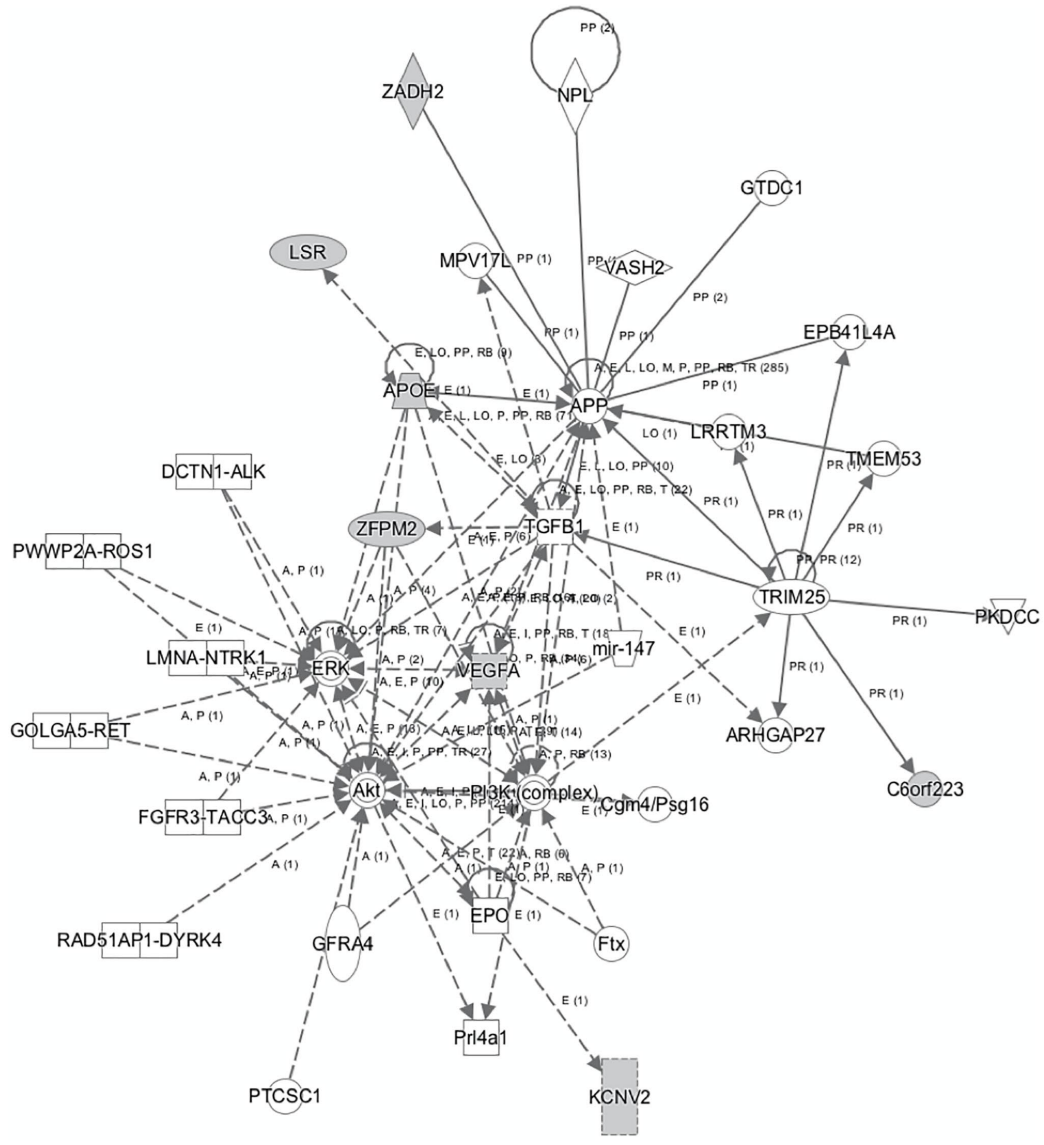
The network that links most of the identified genes that predict AD as generated by IPA tool.

Subsequently, a network linking most of these genes and other mediators was developed using IPA tool (Figure 2). The genes and mediators of this network have functions that correspond to cancer, dermatological diseases and conditions, organismal injury and abnormalities.

The network displayed in Figure 2 shows the multiple associations identified from the analyses, with the *VEGF-A* gene located at the center of many of the observed relationships involved in several metabolic pathways connected to cancer and inflammation. In this context, *VEGF-A* appears to interact with the *EPO, AKT, ERK, TGFB1* genes and the PI3K complex, indicating a potential effect in cellular activities, such as proliferation and apoptosis, where the *AKT, PI3K, ERK* and *TGFB1* cascades and pathways play a significant role. The identified network further enhances the aforementioned notions, by highlighting respective relationships between the *ERK, AKT, PI3K, TGFB1, APP* and the *APOE* gene. In addition, IPA analysis shows direct association between *APP* and the VEGF-A gene, among other associations including the *TGFB1, MPV17L, ZADH2, NPL, VASH2, GTDC1, EPB41L4A, LRRTM3, TRIM25, and ARHGAP27* genes.

## DISCUSSION

Early detection of AD is important for the prognosis of patients, especially to initiate treatments during its pre-symptomatic phase and before the pathological amyloid and tau protein accumulation and the extensive brain damage, which can significantly decrease patient autonomy. Multiple approaches have been used for the early identification of AD-linked parameters including medical assessment, as well as cognitive evaluations and imaging analyses that exploit multimodal biological and molecular features present in AD. Moreover, studies that use regression analyses have demonstrated the relation between AD and variables including clinical examination and cognitive test scores [27]. More recently, artificial intelligence approaches have been used, including supervised predictive analytics tools such as support vector machines, random forests, and artificial neural networks to distinguish AD cases from controls, and for identifying individuals with higher risk of AD in a given period of time [27]. In the present study, we used EN [29], a supervised machine learning method, to assess the role of genetic variants of common AD biomarkers on the prediction of AD risk.

The EN revealed one model with an adequate accuracy of 72% that mostly included epistatic interactions between the assessed variants as predictors of AD risk. The first and strongest genetic predictor was the *APOE e4* allele, which is in agreement with a large volume of scientific results that support this finding [9, 28]. Despite this, the precise processes by which ApoE affects AD remains unclear. AD is characterized by two main features encompassing the existence of extracellular deposition of Aβ generating amyloid plaques, and intracellular occurrence of neurofibrillary tangles (NFT) consisting of clusters of hyper-phosphorylated tau protein. ApoE may be associated with AD through its direct synergism with Aβ proteins. Indeed, the ApoE has been found in Aβ plaques of AD brains [30], and knock-out studies of *APOE* gene in mice have revealed that ApoE is critical for the initiation and retention of Aβ plaques. Furthermore, the direct synergistic link between ApoE and tau protein may also contribute to the association of ApoE and AD. Previously, the existence of ApoE was detected in Tau-NFT mediated deposits [31]. In addition, up-regulation of *APOE4* in neuronal cells of genetically engineered mice triggered a rise in tau hyper-phosphorylation induced by Erk stimulation [6].

Using the EN model, we observed the significant role of the interactions between *APOE* e4 allele polymorphism and the 2 SNPs of the *LSR* gene which we have identified in a previous study [12]. LSR is present in the central nervous system [13] and the *lsr+/−* mice display increased memory deficits following intracerebroventricular injection of the oligomeric soluble form of the β-amyloid peptide [32]. Animal studies have shown the presence of *LSR* gene transcripts in endothelial cells (ECs) of the blood-brain barrier (BBB) [33]. In *lsr−/−* knockout mice, the BBB does not appear to seal during embryogenesis [34], highlighting LSR’s critical role in maintaining BBB integrity. Furthermore, given its role as an ApoE receptor, LSR is involved in lipid metabolism in the brain [13]; Herzine et al, manuscript in preparation) and could, therefore, play a role in AD development, with the present genetic associations further supporting this hypothesis.

The novel results of the present model include five epistatic interactions between VEGF-A-related variants and one APOE*VEGF-A-related polymorphism interaction (rs6993770) associated with decreased risk for AD.

VEGF-A has been proposed as a promising novel therapeutic approach for AD [35]. Transplantation of mesenchymal stem cells into the double transgenic AD mouse model (APPswe/PS1dE9 mutations) leads to improvement of cognitive function [36]. Furthermore, higher VEGF-A concentration in the cerebrospinal fluid has been associated with slower cognitive decline in patients with AD risk [17]. Thus, VEGF-A could be considered as a protective factor for individuals having high risk of AD.

Concerning the interactions between *APOE* and VEGF-A, this is consistent with studies showing that VEGF-A exerts a neuroprotective effect in humanized *APOE ε4* mice, where treatment with VEGF-A leads to improvements of behavioral deficits [37]. A recent study also demonstrated that *APOE ε4* interacts with *VEGF-A* gene expression in the brain to affect cognitive performance [38]. Therefore, VEGF-A alone or in interaction with ApoE, seems to play an important role in AD risk, consistent with the results of this study.

The polymorphism rs6993770 is involved in 2 epistatic interactions with other VEGF-A-related SNPs and one interaction with *APOE ε4* allele. This constitutes an intronic variant of the *ZFPM2* (zinc finger protein, FOG family member 2) gene. The latter codes for the FOG family member 2, which is linked with repression of GATA mediated transcriptional activation [39, 40] and thus with hematopoiesis. The T minor allele has been associated with decreased VEGF-A levels [19, 20]. In the present model, this allele interacted with the non-*ε4* alleles of *APOE* gene to decrease the risk for AD. Also, the TA genotype of rs6993770 interacted with the AA genotype of rs7043199 to decrease the risk for AD. The rs7043199 SNP in an intronic variant of *VLDLR-AS1* gene and is located close to the *VLDLR* gene and its A allele has been associated with decreased VEGF-A levels [19, 20]. The VLDL receptor is a member of the low-density lipoprotein receptor family and binds ApoE. It is involved in pathways essential for the development of laminated structures and for the synaptic plasticity of the brain and is, thus, considered as a receptor that could be involved in the development of AD [41]. It is important to note that this interaction presented the highest coefficient, thus is the strongest predictive factor of the model, with a protective role against AD higher than that of the absence of *e4 APOE* allele. Our team was among the first to identify the allele 4 of the *APOE* gene as the strongest genetic risk factor for AD [8] and in this investigation we are proposing the rs7043199*rs6993770 interaction as a strong protective factor against AD. Finally, the A major allele of the rs6993770 (AA genotype) interacted with the deletion genotype of the rs34528081, which is an intergenic SNP close to *VEGF-A* gene, with the overall result of decreasing the risk for AD. Both alleles of these 2 SNPs have been associated with increased levels of VEGF-A [19, 20]. Thus, this interaction could be linked with further increase in VEGF-A levels that have been shown to have a protective effect against AD [17, 18]. The same deletion genotype of rs34528081 also interacted with the GG genotype of rs2375981, which is an intergenic SNP between *KCNV2* and *VLDLR* gene and whose G allele has been associated with decreased levels of VEGF-A [19, 20]. This interaction that is associated with decreased risk of AD could either be explained by a modification of VEGF-A levels or through an effect on the *VLDLR* gene. It has a very high coefficient in the model, similar to the absence of *APOE ε4* allele, and thus has a strong protective effect. Furthermore, the GC genotype of rs2375981 interacted with the CT genotype of rs2639990 to decrease the risk of AD. This is an intronic variant of *ZADH2* gene and the T allele has been associated with increased levels of VEGF-A [19, 20]. Hypomethylation of differentially methylated positions located on *ZADH2* has been observed in AD patients and have been associated with memory performance and cerebrospinal fluid levels of Aβ and tau [42], thus indicating a role of this gene on AD. Also, the same genotype of this SNP interacted with the GA genotype of rs6921438 to decrease the risk of AD. The rs6921438 is an intergenic SNP located between *LOC100132354* (lnc-RNA) and the *C6orf223* gene (encoding an uncharacterized protein) and is near the *VEGF-A* gene. The A allele is associated with decreased levels of VEGF-A [19, 20] and explains the highest percentage of VEGF-A levels variability (41.19%). This SNP (A allele) has also been associated with decreased HDL and increased LDL [22, 43]. It is thus a marker linked with both VEGF-A levels and lipid metabolism, which could mediate its relationship with AD.

The strengths of the present study include the use of the EN method in the development of the prediction model, which is a machine learning approach more powerful than classical statistics methodologies. Furthermore, the identified model showed a sufficient accuracy of 72% and an AUC of 81%. The accuracy of the model is comparable to other tools [44–46] and this is very important as our model uses genetic factors. In fact, in other studies, the addition of a genetic score led to a small improvement of prediction of the classical variables model [47]. In a recent review, 61 papers describing dementia risk models were identified and most of them had moderate-to-high predictive ability (AUC > 0.70). The highest AUC value was 0.932 [48] and our AUC is 81%. All these data highlight the satisfactory accuracy of the identified prediction model.

A few limitations of the study, however, include the relatively small sample size and the lack of VEGF-A levels’ measurements in the studied populations.

In summary, the prediction model proposed in the present study consists of 8 epistatic interactions that, in combination with the *APOE ε4* allele, directly affect the risk for AD. These interactions involve 9 polymorphisms in 8 genes: *VLDLR-AS1, APOE, KCNV2, ZADH2, C6orf223, LSR, ZFPM2,* and *VEGF-A*.

IPA analysis highlighted relationships between the identified genes and neurological diseases, within the first top five disorders associated with said genes, including CVD and cancer. This finding indicates that the genetic determinants of the selected biomarkers (VEGF-A, LSR and APOE) could act as common links between important chronic diseases. In fact, most of these genes are shown to be linked in the context of an enlarged common network, the functions of which correspond to cancer, dermatological diseases and conditions, organismal injury and abnormalities.

In conclusion, these novel epistatic interactions between *APOE*, *LSR* and VEGF-A related polymorphisms allow for prediction of AD risk, constituting not only a useful prediction model, but also providing new insights about molecular mechanisms that can be involved in AD development which could be useful as biomarkers and/or treatment targets. We are also proposing two epistatic interactions (rs7043199*rs6993770 and the rs2375981*rs34528081) between VEGF-A-related polymorphisms as strong protective factors against AD.

## METHODS

### Population and data collection

The study population consisted of 1078 (602 controls and 476 cases) unrelated adults of European origin, recruited during the period 1996–1998. The study was approved by the related ethics committees and all participants provided written consent prior to their enrollment in the study.

The recruitment and data collection procedures of the present population have been previously extensively described [9]. The clinical diagnosis of AD was based on 5 criteria: the National Institute of Neurological and Communicative Disorders and Stroke and the AD and Related Disorders Association classification of probable AD [28]; the Diagnostic and Statistical Manual of Mental Disorders, Fourth Edition [28]; the International Classification of Diseases and Related Health Problems, 10th Edition [28]; the Mini Mental State Examination score equal to or less than 23 (Mini Mental State Examination >23 for controls) [28]; and, lastly the modified ischemic scale less than three [28]. Secondary causes of dementia were excluded by computerized tomography scan of the brain. Individuals with other chronic or neurological diseases, such as cancer and Parkinson's diseases, respectively, were excluded from the study. Participants serving as controls followed similar assessment and interviews as cases, except for the computerized tomography scan. All controls presented a Mini Mental State Examination >23 and were free of dementia.

Genotyping data were available in a subsample of the population. The final sample size after quality control for genotyping and outliers exclusion was 323 individuals (143 AD cases and 180 controls).

### Genotyping analyses

DNA was extracted from peripheral blood [49] and all samples were stored in biobanks of the BRC IGE-PCV (Biological Resources Center ‘Interactions Gène-Environnement en Physiopathologie Cardio-Vasculaire’ BB-0033-00051). Two SNPs of the *LSR* gene (rs34259399 and rs916147) previously identified as candidate variants for AD were genotyped, along with the 10 VEGF-A-related polymorphisms (rs6921438, rs7043199, rs6993770, rs2375981, rs34528081, rs4782371, rs2639990, rs10761741, rs114694170, rs1740073) and the common *APOE* variants. The genotyping analyses were performed in LGC genomics (http://www.lgcgroup.com) using the competitive allele-specific PCR (KASP) chemistry coupled with a Förster resonance energy transfer-based genotyping system (http://www.kbioscience.co.uk/reagents/KASP/KASP.html). Two of the *APOE* common polymorphisms rs429358 (Cys112Arg) and rs7412 (Arg158Cys) were genotyped as previously described [50].

### Statistical analyses

The agreement of the frequencies of genotypes with the Hardy-Weinberg equilibrium was tested using the chi-squared test.

For the purposes of developing the prediction model, a machine learning method was applied, entitled “Elastic Net” (EN) [29], with the aim of identifying the strongest predictors for the risk of AD combining all genotyping data (direct effects and epistatic interactions), as well as age and sex. In short, the method tests hundreds of logistic regression models and penalizes each of them, in order to reach the final, optimal one. The former constitutes an extensively used method, especially in the development of disease prediction models with special attention to the integration of omics data [51–54]. Furthermore, the method can allow accurate predictions with smaller sample sizes as it tolerates a big number of predictors [29] and it is also used in analyses entailing genetic data, as well as GWAS studies [55, 56] and it is considered to function better in cases where gene * environment interactions are involved in the prediction of a disease [57]. Comparison studies have shown that EN is a powerful tool, especially when additive gene effect is expected [58]. It has also been previously used in AD risk prediction with clinical factors, imaging and omics [59–61] but not to study the effect of candidate genes as predicting factors.

In the context of the present analyses, *APOE* genotypes were divided into 3 groups according to the potential presence of *ε4* allele (coded as 2 = *ε4/4*; 1 = *ε2/4*, *ε3/4*; and 0 = *ε2/2, ε3/2, ε3/3)*. The complete dataset was split into two separate datasets, the “train” dataset that included the 80% of the whole population (AD patients + controls) and the “test” dataset that included the remaining 20% of the whole population (AD patients + controls). This selection was random and was performed by the R command ‘createDataPartition’ that is specific to this. The EN analysis was performed using the R software.

### Pathway analysis

All genes identified to participate in AD risk prediction were further assessed using the QIAGEN Ingenuity Pathway Analysis (IPA) tool, in order to examine potential relationships and identify causal links, with the overall aim of proposing mechanisms to explain the results deriving from the prediction model.
